# Impact of COVID-19 Pandemic on Patients With Rheumatic Diseases in Medina, Saudi Arabia: An Observational Cross-Sectional Study

**DOI:** 10.7759/cureus.60128

**Published:** 2024-05-12

**Authors:** Israa Safar Alsofyani, Basim S Samman, Salem S Alhubayshi, Amjad T Ellahi, Aseel B Alsaedi, Mohammed Almansour

**Affiliations:** 1 Internal Medicine, King Fahad General Hospital, Medina, SAU; 2 Internal Medicine, Ministry of National Guard - Health Affairs, Prince Mohammed Bin Abdulaziz Hospital, Medina, SAU; 3 Medical Specialty, King Fahad Medical City, Riyadh, SAU

**Keywords:** symptom flare-up, vaccination, risk factors, rheumatic diseases, covid-19

## Abstract

Introduction: The Coronavirus disease of 2019 (COVID-19) pandemic undoubtedly ranks among the most health-impacting pandemics throughout medical history. Although the COVID-19 global public health emergency has ended, lessons need to be learned to be more ready to face similar pandemics in the future. Few studies in Saudi Arabia discuss the impact of the COVID-19 pandemic on autoimmune rheumatic disease (AIRD) patients. Thus, this study was conducted to elaborate on the effects of the COVID-19 pandemic on AIRD patients and rheumatology practices in Saudi Arabia.

Methods: This observational cross-sectional study was conducted among patients aged over 14 with AIRD using a pre-designed validated survey questionnaire. Data were collected from AIRD patients who were following up between November 2021 to April 2022 at the Rheumatology Clinic of King Fahad General Hospital in Madinah City, Saudi Arabia. This center was chosen as being the main hospital in the city following patients of AIRD.

Results: A total of 324 patients were included in our study, with the majority (n=264, 81.5%) being females. The mean age was 44.42±14.4 years. Clinical data revealed that 115 (35.5%) of our patients experienced mild COVID-19 infection, 19 (5.9%) suffered from respiratory insufficiency, and seven (2.2%) required admission to the intensive care unit (ICU). Non-compliance to medication was recorded at 25.2%. There were 115 (35.5%) patients who had an AIRD flare that was significantly higher among those who were not adherent to the medications (p<0.001). Disease flare was also significantly seen among patients who were not on prednisone or were on low doses of prednisone (p<0.001). The majority (n=33, 97.1%) of the 34 infected patients who had an AIRD flare had their flare-up at the same time as their COVID-19 infection (p<0.001). COVID-19 vaccination rate was 87.7% (n=284). The most common reason for non-vaccination in 40 (12.3%) patients was the patients’ concern about disease flare-ups by the vaccine or interference of the vaccine with their medication (n=16, 4.9%).

Conclusion: Our study showed a 35.5% (n=115) COVID-19 infection rate. The majority of our AIRD patients sustained minor infections that did not require hospitalization or ICU admission. The majority of the patients who underwent a severe COVID-19 infection course were not on prednisolone or were on low-dose prednisone. Due to COVID-19 restrictions and drug shortages, one in four patients (25.3%) stopped taking their medications and was significantly found to have a high prevalence of underlying AIRD flare. Despite the high vaccination rate, disease flare was the biggest concern for those who were not immunized. Although the COVID-19 pandemic has ended, doctors should be aware of risk factors associated with severe AIRD outcomes that should be balanced based on the infection severity, underlying disease flares, and patient-centered education about medication adherence and vaccination.

## Introduction

Patients with autoimmune rheumatic diseases (AIRD) may require special consideration because receiving immunosuppressant medications makes them more susceptible to viral or bacterial infections [1-3]. The Coronavirus disease of 2019 (COVID-19) pandemic has spread worldwide, and the number of infected patients was increasing every day. About 30-70% of infected patients were asymptomatic; however, 20-50% needed hospitalization [4,5]. Furthermore, the COVID-19 pandemic placed a tremendous burden on AIRD patients to comprehend health-related information about the infection and risk factors linked to a more severe course of COVID-19 infection, improve medication adherence, and make healthy decisions to maintain better outcomes [6-8].

A higher prevalence of COVID-19 infection was reported among AIRD patients than the general population and in patients living in urban areas compared to those living in rural areas [9,10]. The reported prevalence of AIRD patients who tested positive for anti-SARS-CoV-2 Ig antibodies was 2.2% [11]. Furthermore, the majority of AIRD patients who got infected with COVID-19 had mild symptoms, expressed mainly with systemic manifestations and sore throat, while six patients exhibited olfactory dysfunction and gastrointestinal disturbances, and all of them had a favorable disease course and were in remission [12]. Furthermore, the frequency of COVID-19 symptoms was higher among patients with spondyloarthropathy and lower in those on immunosuppressive medication and those compliant with health authority guidance [13]. However, no significant differences in cumulative incidence of COVID-19 and case fatality rate in patients with AIRD compared with the control were found [14].

Increased risk of COVID-19 hospitalization among AIRD patients included older patients having a systemic autoimmune condition [15-17]. Male gender; previous lung disease; glucocorticoid use; and the presence of comorbidities such as cardiovascular disease (CVD), interstitial lung disease and chronic obstructive pulmonary disease (ILD/COPD), chronic kidney disease (CKD), and moderate/high AIRD activity were significantly associated with increased hospitalization in patients with AIRD who got infected with COVID-19 [15-17]. Patients with inflammatory arthritis treated with conventional synthetic and/or biological disease-modifying antirheumatic drugs (DMARDs) have almost the same disease course as the general population when infected with COVID-19 [12]. Adherence rates were higher among those who cited guidelines and conversely lower in those with COVID-19 symptoms [13]. Furthermore, apart from non-adherence, worsened disease activity was associated with patients’ fear and many patients perceived that the worsened disease activity was associated with unplanned healthcare visits, medication non-adherence, difficulty accessing treatment, and difficulty obtaining their AIRD medications during the pandemic [10,18].

One in three patients (36%) with AIRD reported disease flares following COVID-19 infection with higher odds among patients with comorbid conditions including asthma, COPD, diabetes mellitus, and mental health disorders [19,20]. A prevalence vaccination rate of 61.8% to 66.8% for COVID-19 was reported in the literature [20,21]. Vaccination against COVID-19 showed no significant difference compared to non-vaccinated patients with regards to increased risk of flare-up [20-22]. Vaccination hesitancy was not associated with age, gender, education, or comorbidities, and the most reported reason for vaccination hesitancy was fear of side effects [20].

Although the COVID-19 global public health emergency has ended, lessons are to be learned from the impact of the COVID-19 pandemic on AIRD patients so as to be more ready to face any similar pandemics in the future. There were few studies in Saudi Arabia that discussed COVID-19's impact on AIRD patients. Thus, this study was conducted to determine the impact of the COVID-19 pandemic on patients with rheumatic diseases in terms of infection rates, risk factors for hospitalization and ICU admission, medication adherence, AIRD flares, and rate of COVID-19 vaccination and explore the causes of non-vaccination in a Saudi cohort.

## Materials and methods

This was an observational cross-sectional study conducted among AIRD patients aged over 14 years old who were following up between November 2021 and April 2022 at the Rheumatology Clinics of King Fahad General Hospital in Madinah City, Saudi Arabia. This center was chosen as being the main hospital in the city following patients of AIRD. Patients who were less than 14 years old and those who did not have AIRD were excluded from the study. Institutional approval was obtained on the 27th of September, 2021 from the Institutional Review Board (IRB), General Directorate of Health Affairs, Medina, Saudi Arabia (H-03-M-084). 

While patients were waiting for their consultation, all AIRD patients were approached by the interviewers. Formal consent was obtained from the respondents, simply clarifying the aim of the study, the importance of the respondent views, and assuring strict confidentiality of the information. 

Data were collected using a questionnaire consisting of the following four sections: Section 1 contains relevant patient’s sociodemographic characteristics (age and gender), baseline AIRD diagnosis (rheumatoid arthritis, Sjogren’s syndrome, systemic lupus erythematosus (SLE), spondyloarthropathy, systemic sclerosis, inflammatory myopathy, Behçet’s disease, and others), and associated comorbid conditions (diabetes mellitus, CVD, hypertension, lung diseases, liver diseases, CKD, allergic disorder, and others). Section 2 includes questions about the baseline therapy during the COVID-19 pandemic in 2020, such as non-steroidal anti-inflammatory drugs (NSAIDs), methotrexate, sulfasalazine, leflunomide, hydroxychloroquine, Janus kinase (JAK) inhibitors, rituximab, tumor necrosis factor (TNF)-alpha inhibitors, oral/intramuscular steroids, and others. Other parts in this section include questions about the stoppage of any medication by the patient during the pandemic and the reasons for non-compliance. Section 3 contains questions about the history of COVID-19 infection and its outcomes, which include hospitalization, respiratory insufficiency, intensive care unit (ICU) admission, and AIRD flares. Section 4 involves questions about the history of COVID-19 vaccination and reasons for non-vaccination. 

Using the formula for cross-sectional study, n=Z2 P(1-P)/d^2^, where n is the sample size, Z is the statistic corresponding to the level of confidence, P is the expected prevalence, d is the precision and assuming that the expected prevalence is 10%, the calculated sample size was 139 patients [23,24].

Data was analyzed using a licensed Statistical Package for Social Sciences (SPSS) version 23.0 (IBM-SPSS Inc., Armonk, New York, USA). The test of normality of continuous variables was done using the Shapiro-Wilk test. Results were expressed as numbers and percentages (for categorical variables) and as mean and standard deviation (for continuous variables). Correlational analysis was performed using the Chi-square (X2) test and Pearson correlation test. Linear regression analysis was done to determine the significant factors for the increased risk of COVID-19 infection and increased risk of disease flares among AIRD patients.

## Results

A total of 324 patients were included in the study, 60 (18.5%) males and 264 (81.5%) females. The mean age of the patients was 44.42±14.4 years (range: 14 to 80 years old). There were 266 (82.1%) with connective tissue disease, 26 (8.0%) with vasculitis, 24 (7.4%) with spondyloarthropathy, and eight (2.5%) with crystal-induced arthropathy. The most prevalent connective tissue disease diagnosis was rheumatoid arthritis (n=159, 49.1%) followed by SLE in 80 (24.7%) patients (Table 1). There were 116 (35.8%) patients who had comorbid conditions, the most prevalent was hypertension in 45 (13.9%) patients followed by hypothyroidism in 40 (12.3%) patients. 

**Table 1:** Clinicodemographic characteristics of 324 patients with AIRD AIRD, autoimmune rheumatic disease; SD, standard deviation; SLE, systemic lupus erythematosus; ESRD, end-stage renal disease; ILD, interstitial lung disease

Characteristics	Mean (SD)	n=324
Age in years	44.42±14.4	
Gender		
Male		60 (18.5%)
Female		264 (81.5%)
AIRD diagnosis		
Connective tissue disease		266 (82.1%)
Vasculitis		26 (8.0%)
Spondyloarthropathy		24 (7.4%)
Crystal-induced arthropathy		8 (2.5%)
Specific rheumatic diagnosis		
Rheumatoid arthritis		159 (49.1%)
SLE		80 (24.7%)
Behcet’s syndrome		23 (7.1%)
Psoriatic arthritis		18 (5.6%)
Gouty arthritis		8 (2.5%)
Ankylosing spondylitis		6 (1.9%)
Overlap syndromes		6 (1.9%)
Sjogren’s syndrome		5 (1.5%)
Dermatomyositis		5 (1.5%)
Systemic sclerosis		4 (1.2%)
Juvenile idiopathic arthritis		3 (0.9%)
Mixed connective tissue disease		3 (0.9%)
Granulomatosis with polyarthritis		2 (0.6%)
Polyarteritis nodosa		1 (0.3%)
Vogt-Koyanagi-Harada syndrome		1 (0.3%)
Number of patients with comorbidities		116 (35.8%)
Comorbidities		
Hypertension		45 (13.9%)
Diabetes mellitus		31 (9.6%)
Hypothyroidism		40 (12.3%)
Bronchial asthma		6 (1.9%)
Osteoarthritis		12 (3.7%)
Others (ESRD, ILD, anemia, cancer, stroke)		5 (1.5%)

Clinical data showed that 115 (35.5%) of our patients had COVID-19 infection, 19 (5.9%) had respiratory insufficiency secondary to COVID-19 infection, 13 (4.0%) were hospitalized, and seven (2.2%) were admitted to the ICU. There were no significant differences in the proportion of patients who were admitted to the ICU (p=0.856), those who had respiratory insufficiency (p=0.477), those who had mild disease (p=0.818), and those who had COVID-19 according to the different rheumatic conditions (p=0.467). Furthermore, there were no significant differences in the outcomes among patients who had comorbid conditions (p=0.149) (Table 2).

**Table 2:** Proportion of COVID-19 outcomes according to rheumatic diseases in 324 patients ICU, intensive care unit; SLE, systemic lupus erythematosus

Rheumatic diseases	n (%)	Had mild COVID-19 infection, n=115	Respiratory insufficiency, n=19	Admitted to ICU, n=7
Rheumatoid arthritis	159 (49.1%)	52 (32.7%)	10 (6.3%)	4 (2.5%)
SLE	80 (24.7%)	26 (32.5%)	7 (8.8%)	1 (1.3%)
Behcet’s syndrome	23 (7.1%)	11 (47.8%)	1 (5.3%)	1 (4.3%)
Psoriatic arthritis	18 (5.6%)	8 (44.4%)	0	0
Others	44 (13.6%)	18 (40.9%)	1 (2.3%)	1 (2.3%)
P-values		0.467	0.477	0.856

Baseline therapy included conventional DMARDs (n=264, 81.5%), biological DMARDS (n=29, 9.0%), immunosuppressive therapy (n=81, 25.0%), and NSAIDs (n=16, 4.9%). There were 119 (36.7%) patients who were on low-dose prednisone whereas 17 (5.2%) patients were on moderate-dose prednisone. Of the 13 patients who were hospitalized, five patients (38.5%) were not on prednisone, another five patients (38.5%) were on low-dose prednisone, and three (23.1%) were on moderate-dose prednisone (p=0.029). Eight of 19 patients (42.1%) who had respiratory insufficiency were on low-dose prednisone and only three (15.8%) who were on moderate-dose prednisone had respiratory insufficiency (p=0.070). Of the seven patients who were admitted to the ICU, three (42.9%) were not on prednisone, three (42.9%) were on low-dose prednisone, and only one patient (14.3%) was on moderate-dose prednisone (p=0.308) (Table 3).

**Table 3:** Outcome of COVID-19 infection in 324 patients according to prednisone doses ICU, intensive care unit

Outcomes	Not on prednisone	On low-dose prednisone	On moderate-dose prednisone	p-values
Respiratory insufficiency (n=19)	8 (42.1%)	8 (42.1%)	3 (15.8%)	0.070
ICU admission (n=7)	3 (42.9%)	3 (42.9%)	1 (14.3%)	0.308
Rheumatic disease flare (n=115)	52 (45.2%)	50 (43.5%)	13 (11.3%)	<0.001

**Figure 1:**
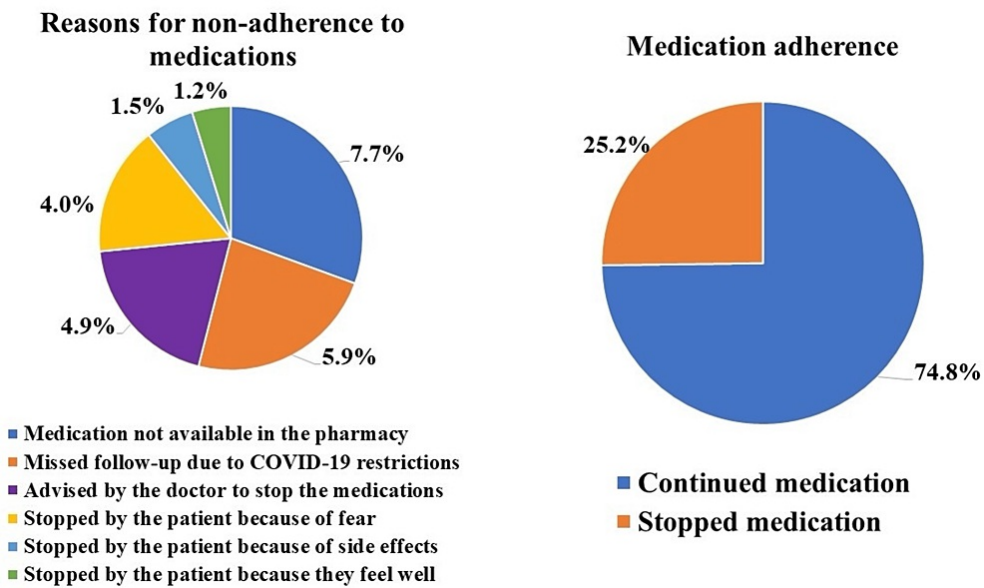
Adherence to medications and reasons for non-adherence

The most commonly prescribed medication was hydroxychloroquine (n=194, 59.9%). Eighty-two patients (25.3%) stopped medications, whereas 242 patients (74.8%) continued taking their medications. The most common reason for non-compliance to medication was that the medication was not available in the pharmacy (n=25, 7.7%) (Figure 1).

There were 115 (35.5%) patients who had AIRD flares. The proportion of patients who had disease flare-ups was significantly higher among those who were not adherent to the medications (n=52, 63.4%) compared to those who continued taking their medications (n=63, 26.0%) (p<0.001). Thirty-three out of the 34 (97.1%) flared-infected patients had their AIRD flare-up during their COVID-19 infection (p<0.001). Disease flare was also significantly seen among patients who were not on prednisone (n=52, 45.2%) and also those who were on low-dose prednisone (n=50, 43.5%) (p<0.001) (Table 3).

**Figure 2:**
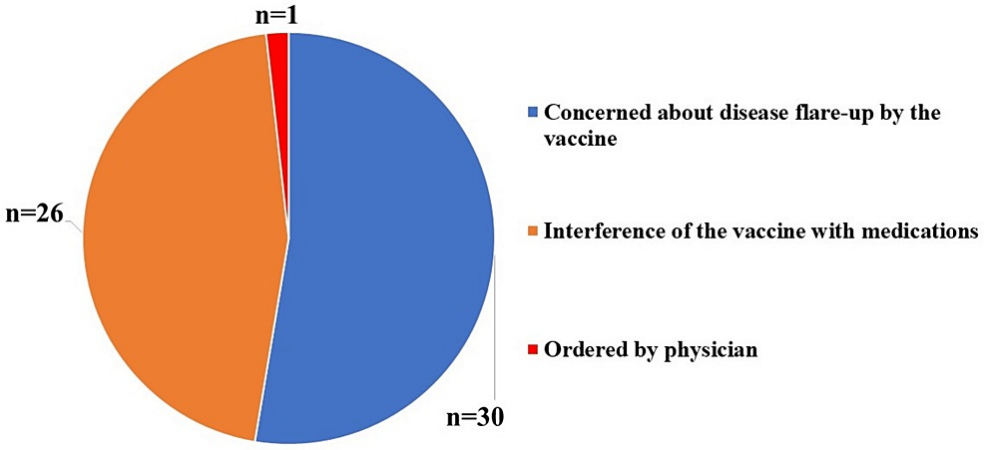
Reasons for not having the COVID-19 vaccine

The COVID-19 vaccination rate was recorded at 87.7% (n=284 patients). The most common reason for non-vaccination in 40 (12.3%) patients was the patients’ concern about disease flare-ups by the vaccine or interference of the vaccine with their medication (n=16, 4.9%) (Figure 2).

A linear regression analysis of factors that were significantly correlated with stoppage of taking medication showed that any rheumatic disease flare-up during the pandemic was the most significant factor for stoppage/non-adherence (beta=0.359, 95% CI=0.229 to 0.423, p<0.001) and history of COVID-19 (beta=-0.088, 95% CI=-0.176 to 0.017, p=0.106). 

## Discussion

In this study, we presented a COVID-19 infection incidence among AIRD patients (35.5%, n=115), with a 7.1% (n=26) severe infection outcome rate; the majority of the patients had mild COVID-19 infection. Despite the fact that the studied patients' infection rates were rather high, the proportion of AIRD patients who had severe disease was significantly lower than the 10.3% reported by Bakasis et al. in 2021 [22]. According to studies, persons with AIRD are more likely to get COVID-19 due to the prevalence of concomitant conditions, the use of glucocorticoids, and high disease activity [23-25].

Studies show that the risk of hospitalization and mortality may be modulated by certain treatments such as corticosteroids and can be negatively impacted by a high dosage of systemic corticosteroids but not by DMARDs [26,27]. This contrasts with the results of the current study that found that a higher proportion of the patients who were not on prednisone or were on low-dose prednisone had a more severe infection outcome, which could be attributed to their underlying AIRD flare during their COVID-19 infection. This result was supported by the fact that our study found that 33 out of the 34 (97.1%) flared-infected patients had their AIRD flare-up during their COVID-19 infection (p<0.001). Additional future studies of larger populations and in more expanded geographic areas should be considered to determine further reasons why AIRD patients in our study who were not on prednisone or were on low-dose prednisone had a more severe COVID-19 infection outcome.

Our study reported a 25.3% (n=82) stoppage of medication. The most common reason for non-compliance was that the medication was not available in the pharmacy (n=25, 7.7%). Studies suggested that individuals with AIRD who develop mild COVID-19 symptoms should stop taking immunomodulating medications for one to three weeks after the onset of the illness, and individuals with rheumatic disease who have positive SARS-CoV-2 test results and risk factors for poor outcomes should stop taking immunomodulating medications and consider antiviral treatment [23]. Patients receiving prolonged immunosuppressive therapy become concerned about their compromised immune response system, which may be a big contributor to the stoppage of their medications [28]. However, some of the most commonly administered medications such as hydroxychloroquine were reported to be effective and acceptably safe for treating SARS-CoV-2-related pneumonia, as shown by the results of recent Chinese clinical trials [29]. 

The vaccination rate was relatively high among the patients in this study (87.7%, n=284), higher than reports from other studies [20,21]. When disease activity is under control and there are no concurrent infections, patients with AIRDs should get the COVID-19 vaccine [30]. COVID-19 vaccines typically induce antibody responses in individuals with managed rheumatic disease, although medications such as mycophenolate and B-cell-depleting treatments have a significant risk of ineffective responses [23].

As the study was based on questionnaires that were completed by interviewing AIRD patients, the possible bias in their responses is this study's potential limitation. Additionally, we were unable to collect several laboratory results to confirm actual disease flare-ups. However, we were able to give every participant a uniform input; therefore, a high level of dependability was attained to represent the percentage of patients who were infected with COVID-19.

## Conclusions

Our study showed a 35.5% (n=115) COVID-19 infection rate, the majority of which were mild infections that did not necessitate hospitalization or ICU admission. The majority of the patients who underwent a severe COVID-19 infection course were not on prednisolone or were on low-dose prednisone. Moreover, 33 out of the 34 (97.1%) flared-infected patients had their AIRD flare-up during their COVID-19 infection. Due to COVID-19 restrictions and drug shortages, one in four patients (25.3%) stopped taking their medications. Despite the high vaccination rate, the biggest concern was a disease flare-up among those who were not immunized. Although the COVID-19 global public health emergency has ended, and even the majority of AIRD patients had a mild COVID-19 infection, lessons are needed to face similar pandemics in the future. For this reason, doctors should be aware of the risk factors of severe AIRD outcomes that should be balanced based on the infection severity, underlying disease flares, and patient-centered education about medication adherence and vaccination. 

## References

[1] Furer V, Rondaan C, Heijstek MW (2020). 2019 update of EULAR recommendations for vaccination in adult patients with autoimmune inflammatory rheumatic diseases. Ann Rheum Dis.

[2] Westra J, Rondaan C, van Assen S, Bijl M (2015). Vaccination of patients with autoimmune inflammatory rheumatic diseases. Nat Rev Rheumatol.

[3] Pablos JL, Galindo M, Carmona L (2020). Clinical outcomes of hospitalised patients with COVID-19 and chronic inflammatory and autoimmune rheumatic diseases: a multicentric matched cohort study. Ann Rheum Dis.

[4] Dreher M, Kersten A, Bickenbach J (2020). The characteristics of 50 hospitalized COVID-19 patients with and without ARDS. Dtsch Arztebl Int.

[5] Gabutti G, d'Anchera E, Sandri F, Savio M, Stefanati A (2020). Coronavirus: Update related to the current outbreak of COVID-19. Infect Dis Ther.

[6] Ferri C, Giuggioli D, Raimondo V (2020). COVID-19 and rheumatic autoimmune systemic diseases: report of a large Italian patients series. Clin Rheumatol.

[7] Abualfadl E, Ismail F, Shereef RR (2021). Impact of COVID-19 pandemic on rheumatoid arthritis from a multi-centre patient-reported questionnaire survey: influence of gender, rural-urban gap and north-south gradient. Rheumatol Int.

[8] Hyrich KL, Machado PM (2021). Rheumatic disease and COVID-19: epidemiology and outcomes. Nat Rev Rheumatol.

[9] Migkos MP, Kaltsonoudis E, Pelechas E (2021). Use of conventional synthetic and biologic disease-modifying anti-rheumatic drugs in patients with rheumatic diseases contracting COVID-19: a single-center experience. Rheumatol Int.

[10] Murray K, Quinn S, Turk M (2021). COVID-19 and rheumatic musculoskeletal disease patients: infection rates, attitudes and medication adherence in an Irish population. Rheumatology (Oxford).

[11] Mena Vázquez N, Manrique-Arija S, Cabezudo-García P (2021). Incidence and case fatality rate of COVID-19 in patients with inflammatory articular diseases. Int J Clin Pract.

[12] Hassen LM, Almaghlouth IA, Hassen IM (2020). Impact of COVID-19 outbreak on rheumatic patients' perceptions and behaviors: a cross-sectional study. Int J Rheum Dis.

[13] Freites Nuñez DD, Leon L, Mucientes A (2020). Risk factors for hospital admissions related to COVID-19 in patients with autoimmune inflammatory rheumatic diseases. Ann Rheum Dis.

[14] Montero F, Martínez-Barrio J, Serrano-Benavente B (2020). Coronavirus disease 2019 (COVID-19) in autoimmune and inflammatory conditions: clinical characteristics of poor outcomes. Rheumatol Int.

[15] Hasseli R, Mueller-Ladner U, Hoyer BF (2021). Older age, comorbidity, glucocorticoid use and disease activity are risk factors for COVID-19 hospitalisation in patients with inflammatory rheumatic and musculoskeletal diseases. Rmd Open.

[16] Bakasis AD, Mavragani CP, Boki KA (2021). COVID-19 infection among autoimmune rheumatic disease patients: data from an observational study and literature review. J Autoimmun.

[17] Grainger R, Kim AH, Conway R, Yazdany J, Robinson PC (2022). COVID-19 in people with rheumatic diseases: risks, outcomes, treatment considerations. Nat Rev Rheumatol.

[18] Xu C, Yi Z, Cai R, Chen R, Thong BY, Mu R (2021). Clinical outcomes of COVID-19 in patients with rheumatic diseases: a systematic review and meta-analysis of global data. Autoimmun Rev.

[19] D'Silva KM, Jorge A, Cohen A, McCormick N, Zhang Y, Wallace ZS, Choi HK (2021). COVID-19 outcomes in patients with systemic autoimmune rheumatic diseases compared to the general population: a US multicenter, comparative cohort study. Arthritis Rheumatol.

[20] Zen M, Fuzzi E, Astorri D (2020). SARS-CoV-2 infection in patients with autoimmune rheumatic diseases in northeast Italy: a cross-sectional study on 916 patients. J Autoimmun.

[21] Grange L, Guilpain P, Truchetet ME, Cracowski JL (2020). Challenges of autoimmune rheumatic disease treatment during the COVID-19 pandemic: a review. Therapie.

[22] Lakota K, Perdan-Pirkmajer K, Hočevar A, Sodin-Semrl S, Rotar Ž, Čučnik S, Žigon P (2020). COVID-19 in association with development, course, and treatment of systemic autoimmune rheumatic diseases. Front Immunol.

[23] Shin YH, Shin JI, Moon SY (2021). Autoimmune inflammatory rheumatic diseases and COVID-19 outcomes in South Korea: a nationwide cohort study. Lancet Rheumatol.

[24] Au K, Reed G, Curtis JR, Kremer JM, Greenberg JD, Strand V, Furst DE (2011). High disease activity is associated with an increased risk of infection in patients with rheumatoid arthritis. Ann Rheum Dis.

[25] Serling-Boyd N, D'Silva KM, Hsu TY (2021). Coronavirus disease 2019 outcomes among patients with rheumatic diseases 6 months into the pandemic. Ann Rheum Dis.

[26] Deepak P, Kim W, Paley MA (2021). Effect of immunosuppression on the immunogenicity of mRNA vaccines to SARS-CoV-2: a prospective cohort study. Ann Intern Med.

[27] Furer V, Eviatar T, Zisman D (2021). Immunogenicity and safety of the BNT162b2 mRNA COVID-19 vaccine in adult patients with autoimmune inflammatory rheumatic diseases and in the general population: a multicentre study. Ann Rheum Dis.

[28] Danza A, Ruiz-Irastorza G (2013). Infection risk in systemic lupus erythematosus patients: susceptibility factors and preventive strategies. Lupus.

[29] Colson P, Rolain JM, Lagier JC, Brouqui P, Raoult D (2020). Chloroquine and hydroxychloroquine as available weapons to fight COVID-19. Int J Antimicrob Agents.

[30] Hazlewood GS, Pardo JP, Barnabe C (2021). Canadian Rheumatology Association recommendation for the use of COVID-19 vaccination for patients with autoimmune rheumatic diseases. J Rheumatol.

